# Weight Gain in HIV Adults Receiving Antiretroviral Treatment: Current Knowledge and Future Perspectives

**DOI:** 10.3390/life14111367

**Published:** 2024-10-24

**Authors:** Konstantinos Markakis, Olga Tsachouridou, Eleni Georgianou, Dimitrios Pilalas, Sideris Nanoudis, Symeon Metallidis

**Affiliations:** Infectious Diseases Division, 1st Internal Medicine Department, AHEPA University Hospital, 54636 Thessaloniki, Greece; conmark@windowslive.com (K.M.); elenigeorgi@hotmail.com (E.G.); sidnanoudis@yahoo.gr (S.N.); metallidissimeon@yahoo.gr (S.M.)

**Keywords:** HIV, weight gain, antiretroviral agents, obesity, ART switch

## Abstract

Body weight is impacted by several individual host and environmental factors. In a person living with HIV (PLWH), weight is also influenced by the disease stage. Wasting syndrome is derived from disease progression, and it can be reversed by the effective use of highly active antiretroviral therapy (HAART). Body weight alterations have been studied and compared in several clinical ART trials, and they differ according to antiviral regimens. The newer integrase strand transfer inhibitors (INSTIs), such as bictegravir and dolutegravir, especially when co-administered with tenofovir alafenamide fumarate (TAF), seem to lead to greater weight increases compared to regimens that include tenofovir disoproxil fumarate (TDF), which seem to have an attenuating effect on weight gain. Nevertheless, despite the established association between INSTI and TAF and the negative impact on weight, more recent data suggest a more cautious approach when HAART treatment decisions are taken. In this manuscript, we review weight changes among PLWH receiving HAART and the relevant underlying pathogenic mechanisms described in recent literature. We try to provide a more critical appraisal of the available data and to underline the challenges in assessing the role of HAART in weight changes in both ART initiation and setting switching.

## 1. Introduction

Since the introduction of the highly active antiretroviral treatment (HAART), the management of people living with human immunodeficiency virus (PLWH) has changed fundamentally. The administration of HAART has transformed a deadly infection into a chronic condition. As a result, the incidence of fatal complications of acquired immunodeficiency syndrome (AIDS), like malignancies and opportunistic infections, has declined dramatically, and PLWH have a nearly normal life expectancy when diagnosed and treated properly [1,2,3,4]. The prolonged life expectancy, due to successful HAART implementation, has resulted in an ever-aging PLWH population where CVD-related morbidity and mortality are growing problems [5,6,7].

Several studies have shown that PLWH have an increased prevalence of CVDs compared to seronegative controls [8,9]. More specifically, PLWH have an increased risk of acute myocardial infarct [10,11,12], heart failure and stroke [13,14]. HIVper se, traditional and non-traditional risk factors are implicated in the increased prevalence of CVD in PLWHs [15]. Except that, cumulative data support that HIV infection can be a cause of atrial fibrillation (AF) in PLWHs [16]. A high viral load and low CD4+ lymphocyte levels have been identified as risk factors for AF [17]. As to the pathogenetic mechanism, ectopic triggers located in sites other than the pulmonary veins seem to be the cause of AF in many PLWHs [18]. Nevertheless, a study on African HIV-positive men challenges the relationship between HIV infection and AF, as the incidence was similar to healthy controls [19]. As lifelong treatment with antiretrovirals is needed to suppress HIV, PLWH face the long-term consequences of HAART treatment [20]. Weight gain and consequent metabolic syndrome (MetS), increased cardiovascular disease (CVD), diabetes, and non-alcoholic fatty liver disease risk are among the most common side effects of HAART. Those effects, in turn, contribute to increased morbidity and, consequently, mortality in an ever-aging patient population [21]. Recently, weight gain in PLWH has been associated with increased risk of diabetes mellitus, MetS, and adverse cardiovascular events, even in the first year after HAART initiation [22].

Weight gain has been one of the first metabolic changes described in patients receiving HAART. Over the last decades, the incidence of obesity has increased in PLWH [23] and is nowadays higher than that in the general population [24]. HAART, viral-associated factors, sex, and ethnicity have been related to weight increase in those patients [25]. Antiretroviral drugs, especially the first ones, used as part of HAART have a profound negative impact on the patients’ metabolism, with high rates of dyslipidemia, lipodystrophy, weight gainand obesity. Those side effects have been, in turn, associated with a risk of increased insulin resistance, diabetes, CVD, and coronary heart disease (CAD) [26,27,28,29,30,31]. However, the effect on weight gain is not the same for every antiretroviral agent, and both interclass and within-class differences regarding the metabolic impacts and grades of weight gain of those regimens have been documented. Some newer agents demonstrate a more profound impact on body mass index (BMI) than older ones [32,33]. Moreover, in the last few years, a new INSTI, bictegravir (BIC), as well as two-drug combinations and long-acting combinations have entered clinical practice, offering an additional therapeutic alternative for PLWHs [34,35,36,37,38,39]. A switch from older to contemporary agents and drug combinations is a promising approach for addressing weight gain and obesity in PLWHs. The purposes of this review are to offer a synopsis of the pathogenetic mechanisms of HAART-induced weight changes in PLWH based on contemporary evidence, to summarize current knowledge on the risk factors linked to weight gain in PLWH, to report on the effects of individual antiretroviral agents on weight, to summarize clinical evidence on the effect of a policy switch from older to contemporary treatment options on weight disorders, and to offer possible approaches to address this important health issue.

## 2. Materials and Methods

This is a narrative review. We searched Pubmed/Medline for articles using the following keywords in the literature search: (“HIV”, “antiretroviral treatment”, or “HAART”) in combination with the terms (“weight gain” and “obesity” or “metabolic syndrome”). We focused on observational studies, retrospective studies, clinical trials, randomized control trials (RCTs), guidelines, meta-analyses, and reviews; other similar types of articles were also eligible for this review. Articles published until September 2024 were eligible.

## 3. Pathogenetic Pathways of HAART-Induced Weight Disorders in HIV Infection

In the pre-HAART era, weight loss was one of the main characteristics of HIV infection. The mechanism of weight gain in PLWH remains largely unclear. While in patients with advanced disease, weight gain reflects immune reconstitution and is linked with reduced mortality; weight increase in patients who were already obese has been linked with increased CVD and diabetes risk [40,41]. Patients with lower CD4 baseline levels and higher viral load experience larger increases, as previous studies demonstrated [40]. This phenomenon may be attributed to the return to normal function, which is a consequence of immune reconstitution following HAART initiation [42].

Several studies highlight the impact of antiretroviral regimens on adipocytes and cytokines involved in glucose and lipid metabolism [43,44,45,46,47,48,49]. More specifically, HAART promotes the expression of genes involved in the formation of adipocytes in fibrosis and hypoxia, and affects the metabolic and immune profile of adipocytes in animal models infected with the Simian Immunodeficiency Virus (SIV) [50].

The mechanisms of adipogenesis are better studied in INSTIs. INSTIs act directly on adipose tissue, hindering the process of fat beiging, which is connected to beneficial metabolic effects and weight loss [32]. Antiretroviral drugs associated with significant weight gain, like dolutegravir (DTG) or raltegravir (RAL), as well as BIC and elvitegravir (ELV), promote the fibrosis of adipose tissue (AT) and the accumulation of lipids in adipocytes, and are associated with increased adipogenesis compared to controls [51,52]. Similarly, the exposure of adipocytes derived from macaques to INSTIs promotes the hypertrophy and fibrosis of AT, inhibits white AT beiging capacity, and leads to a hypoxic environment, which in turn further promotes fibrosis [53]. Domingo et al. demonstrated that INSTIs’ effect on adipocyte function takes place through interference in the repression and expression of adipokine and cytokine genes rather than by affecting adipocyte differentiation [54]. However, other studies support the interference of INSTIs in the adipocyte differentiation process [44]. In particular, DTG inhibits the release of adipokine and leptin and increases the release of proinflammatory cytokines [55]. In another study, the switch from boosted darunavir (DRV) to DTG-containing regimens resulted in decreased adiponectin release, highlighting the effects of antiretroviral treatment on lipid and glucose metabolism [56]. NNRTIs also interfere with the release of adiponectins. Efavirenz demonstrates a proinflammatory and anti-adipogenic reaction, whereas nevirapine has a neutral effect on adipogenesis [57]. Rilpivirine (RPV) also demonstrates an efavirenz-like effect, although only in high concentrations [46]. Compared to lopinavir/ritonavir, efavirenz (EFV) decreases the expression of PPARγ and adiponectin, promotes the release of proinflammatory cytokines, and reduces adipokine release to a much greater grade [58]. Likewise, Moure et al. demonstrated that agents with neutral or less profound impacton weight gain, like EFV and ELV, respectively, may interfere with the adipocyte differentiation and promote the release of proinflammatory cytokines, inhibiting the expression levels of adipogenesis marker genes [44].

Whether weight gain observed in INSTI-based treatments is caused by the more rapid viral load decline, and a faster return to a normal process is not clearly answered. Nevertheless, some longitudinal studies that assessed weight gain in different time points have shown significant differences in weight increases between DTG and non-DTG regimens at time points where the grade of viral suppression is equal [59,60].

Changes in the composition of gut microbiota seem to play a significant role in the pathogenesis of MetS and weight gain in PLWH. Even more, gut microbiota and the presence of enteric dysbiosis can affect the immune response to HAART [61]. Gut microbiota in PLWH is significantly less diversified than that in healthy controls [62,63]. Interestingly, elite controllers have amuch richer gut microbiota resembling that of healthy controls [64,65]. On the contrary, seronegative infants of HIV-positive women with documented exposure to the virus exhibited pathological changes in gut microbiota, thus underlining the significant role of HIV in enteric dysbiosis [66]. Recently, Ishizaka et al. linked weight gain in PLWH with the loss of a specific bacteria, the Parabacteroides [67]. Enteric dysbiosis, increased enteric permeability, and consequent inflammation are found in untreated as well as in treatment-experienced PLWHs [62,67]. However, the grade of dysbiosis is different depending on the antiretroviral regimens used, as previous studies demonstrated that antiretroviral agents have different impact on gut microbiome [63,68,69]. Recently, Narayanan et al. studied the impact of different antiretroviral drugs on the oral and gut microbiota. They found that the different antiretroviral regimens are related to significantly distinct microbial content. In PLWH receiving INSTI-based HAART, Faecalibacterium and Bifidobacterium are much more abundant, while NNRTIs are related to an abundance of Escherichia coli, Gordonibacter, and other bacteria [63]. A study comparing the impact of INSTI and PI-based treatment on microbiota demonstrated significant differences between the two [70]. More specifically, enteric dysbiosis is more frequent, with a decreased short-chain-fatty-acid-producing species and bacterial diversity in patients receiving INSTIs. Considering the relationship between gut dysbiosis and obesity, this could offer a possible explanation of the link between INSTI-based treatment and weight gain. However, pathways implicated in the production of lipopolysaccharide compounds are increased. Increased intestinal permeability and microbial translocation resisting even after HAART initiation lead to disturbed gut function and consequently to chronic inflammation and modified metabolom which are important pathogenetic mechanisms of weight gain in PLWHs [71].

Another proposed theory suggested that the interference of antiretroviral drugs with the melanocortin signaling system results in weight gain in a way similar to that observed in psychiatric patients receiving antipsychotic medication [72]. However, this mechanism has been rejected by the consequent work of McMahon [73]. In this in vitro study with cell-based assays, INSTIs demonstrate an antagonistic action on the human melanocortin 4 receptor, which is central in the regulation of the feeding behavior; however, this action only occurs in concentrations significantly higher than the expected clinical exposure.

As for the mechanism explaining the excess weight gain when switching from TDF to TAF or from efavirenz to INSTIs, it seems that TDF and EFV have weight-suppressing effects, which are lost after switching the treatment [74]. However, whether TDF-induced weight loss or TAF-induced weight gain is the main pathophysiological mechanism remains elusive, and more data are needed [75].

PIs may promote weight gain through a variety of pathophysiological pathways, which include disturbed glucose and lipid metabolism, increased pro-inflammatory cytokines release, mitochondrial toxicity, and PI-induced lipodystrophy [76].

**Figure 1:**
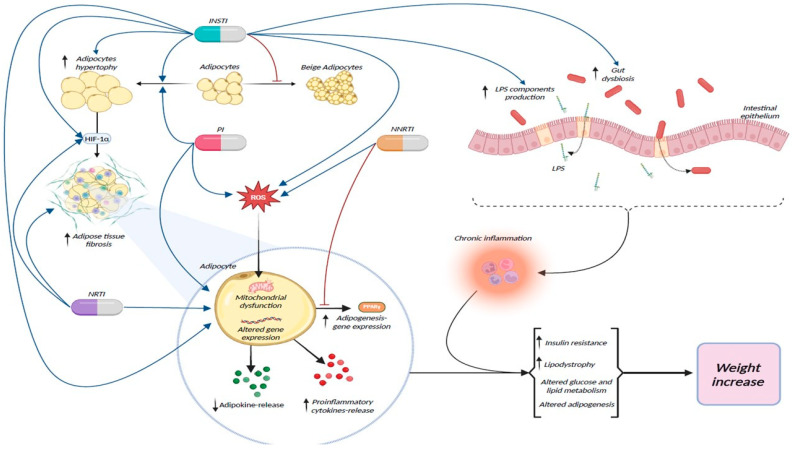
Pathogenetic mechanisms associated with antiretroviral treatment and weight gain. Antiretroviral drugs have the potential to interfere with the metabolism of adipocytes through a variety of mechanisms. INSTIs inhibit the process of adipocyte-being, which has a beneficial effect and is linked to weight loss. Moreover, INSTIs and PIs promote the hypertrophy of adipocytes, which in turn leads to increased fibrosis of adipose tissue through a hypoxia-driven process with the interference of the HIF-1α factor. NRTIs (especially TAF) have also been linked to increased hypoxia and fibrosis of adipocyte tissue. Through the increased release of ROS, INSTIs, and NNRTIs impair the function of mitochondria in adipocytes. Additionally, HAART interferes with the expression of genes that regulate adipogenesis through factors like PPARγ and the release of cytokines and adipokines. Those HAART effects on adipocytes lead to a diminished release of adipokines like adiponectin and leptin, an increased release of proinflammatory cytokines, and a modified expression of genes associated with adipogenesis (with some agents like NNRTIs inhibiting and others like some INSTIs promoting adipogenesis). As a result, increased insulin resistance, pathological glucose and lipid metabolism of the adipocytes, and lipodystrophyare promoted, especially with older PIs. INSTIs have also been linked to enhanced gut dysbiosis, which, in combination with the increased intestinal permeability observed in people with HIV, leads to the intensified microbial translocation and circulation of microbial products like lipopolysaccharide and results in a state of chronic inflammation. The combination of those factors, among potential others that remain elusive, contributes to the weight gain observed in people receiving HAART. Created with BioRender (© 2024 BioRender, Toronto, ON, Canada). Abbreviations: AT: adipose tissue; cART: combined antiretroviral treatment; HIF-1α factor: hypoxia-inducible factor 1-alpha; INSTI: integrase strand transfer inhibitor; NRTI: nucleoside reverse transcriptase inhibitor; NNRTI: non-nucleoside reverse transcriptase inhibitor; PI: protease inhibitor; PPARγ: peroxisome proliferator-activated receptor gamma; ROS: reactive oxygen species; TAF: tenofovir alafenamide; blue arrows: promoting action of antiretroviral agents; red arrows: inhibitory action of antiretroviral agents.

Lastly, the excess weight increase observed in patients receiving modern-era antiretroviral agents compared to older agents could partly be explained by less frequent adverse effects, like gastrointestinal side effects, observed in patients treated with contemporary regimens [42,75]. The relevant pathogenic mechanisms associated with weight gain are illustrated in Figure 1.

## 4. Risk Factors Associated with HAART-Induced Weight Gain

In previously underweight patients, weight gain after HAART initiation represents a return to a normal weight as part of the immune reconstitution [40]. Furthermore, the pretreatment BMI determines the magnitude of the survival benefit caused by HAART, as weight gain is linked to a low mortality post-HAART-initiation only in patients who were not overweight. The Data Collection on the Adverse Events of Anti-HIV Drugs (D:A:D) cohort study demonstrated that in PLWH with normal pretreatment BMI, weight gain after HAART initiation is associated with adverse long-term effects and increased cardiovascular disease (CVD) risk [27]. Except for increased CVD risk, weight gain in the first year after HAART initiation has been found to significantly increase the risk of diabetes mellitus in PLWH [27,28,41]. Moreover, the relative risk of developing diabetes with weight gain is significantly greater than that in uninfected individuals. The prodiabetic effect varies between the different antiretroviral drug classes. In particular, initiating treatment with a raltegravir (RAL) or a PI drug combination is associated with a substantially greater risk of diabetes mellitus compared to combinations with NNRTI [31]. This adverse effect is only partially attributed to weight gain in the first twelve months. In patients receiving an INSTI-based regimen, a weight gain of over 5% of the baseline body weight has been linked with the emergence of insulin resistance as determined by the homeostasis model assessment of insulin resistance (HOMA-IR) [77].

Except for HAART-specific characteristics, host-risk factors, like sex, origin, baseline BMI, and CD4 T-cell levels, as well as genetic factors, also contribute to antiretroviral-mediated weight gain. Specific mitochondrial DNA haplogroups are associated with greater BMI increases in patients switching to INSTI regimens [78]. Preswitch-status also influences the risk of weight gain, with PLWH who have low CD4 levels before HAART initiation being at more risk of weight increase after starting a DTG-based therapy [79]. Moreover, the combinations of DTG with TAF/FTC or TDF/FTC as backbones had greater impacts on weight than the ABC/LAV combination.

Several studies demonstrate that PLWH born female face a greater risk of weight gain when on INSTI-regiments [80,81,82], though in other studies female sex is associated with a reduced risk of weight increase [79]. In addition, hormonal status also seems to influence weight gain. Assoumou et al. demonstrated that women with ovarian reserves, defined as the detectable anti-Müllerian hormone, are protected from weight gain and the development of insulin resistance after switching to RAL/ETV [83]. In contrast, postmenopausal women, as well as men, are prone to weight gain and associated insulin resistance after the switch. However, another study by Hamzah, where premenopausal and postmenopausal women are switched from TDF/FTC/NNRTI to ABC/3TC/DTG, does not confirm the protective role of ovarian function against weight gain and insulin resistance [84]. A retrospective study of over 300 pregnant seropositive women on HAART supports that the risk of excess gestational weight gain is significantly increased among those receiving TAF/INSTI combinations, whereas pregnant women on TDF have decreased risks [85].

In conclusion, possible risk factors of excessive weight gain caused by HAART include host-specific characteristics like sex, origin, pretreatment BMI, and CD4 baseline, as well as the genetic factors and hormonal status of the patient. Nevertheless, for many of those factors, the available data are contradictory.

## 5. Antiretroviral Class-Specific Effects on Weight

### 5.1. NRTIs

Tenofovir is used as the NRTI backbone in most triple antiretroviral combinations. Tenofovir is available in two pharmacological forms: TDF and tenofovir alafenamide (TAF). TAF is less nephrotoxic, and its effect on bone density is smaller than TDF’s, hence leading to a treatment switch from TDF to TAF in a vast number of patients. However, since then, concerns have been raised about the effect of TAF on weight. In a cohort of over 6900 patients, the switch from a TDF-based regimen to a TAF-based regimen leads to a significant weight increase, irrespectively of the other antiretrovirals contained in the ART regimen [86]. In a clinical trial comparing TDF to TAF combination treatments, TAF results in a significantly higher weight increase [87]. This weight gain is even more obvious in female patients and in combination with the DTG. Similarly, PLWH of the ATHENA Dutch observational study who switch from TDF to TAF experience significant weight increas, particularly females and PLWH starting INSTI simultaneously with TAF [88]. In PLWH, where a simultaneous switch to TAF and INSTI was performed, over 10% weight gain was observed in 14% of the patients. Besides confirming the independent association of TAF and INSTI combinations and weight gain in PLWH who switch regimens, Palella et al. demonstrate that BMI increase is mainly observed in the first eight months post-switch and is INSTI-driven, whereas afterward, TAF is responsible for the consequent recorded weight increase [89]. In patients on RPV-based regimens, the switch from TDF/FTC/RPV to TAF/FTC/RPV is also associated with weight increase [90]. Similarly, further prospective and retrospective studies confirm the impact that switching from TAF to TDF has on BMI, especially in older patients and those receiving HAART for a long time period [91,92,93]. Hence, TAF has the same impact on weight gain in ART-experienced patients as in treatment-naïve patients. The effect of TAF on weight is also greater than that of two other NRTIs: abacavir (ABC) and zidovudine [94]. In the RESPOND study, the TAF is associated with a more than 7% BMI increase compared to lamivudine-based ART [95]. This association was more profound when TAF and DTG were co-administered.

Inversely, in a South African randomized controlled trial, switching from TAF/FTC/DTG to TDF/3TC/DTG results in significant weight loss in women, not in men [96].

### 5.2. NNRTIs

Giacomelli et al. studied the impact of switching to a new ART regimen on weight uptake, CVD risk, and lipid levels among virologically suppressed PLWH. Whereas switching to the combination of TAF/FTC/RPV or TAF/FTC/EVG is associated with a significant weight increase, the ABC/3TC/DTG combination does not seem to have a negative effect [97]. This comes in contrast to other studies that demonstrate that DTG is strongly linked to BMI increase. However, this can partly be explained by the presence of TAF, in the first two combinations, which is known for this property.

### 5.3. PIs

PIs combined with double NRTIs have been one of the first drug combinations approved for treating HIV-infected adults. Despite being efficient, first-generation PIs frequently lead to dyslipidemia and lipodystrophy. The effects of PIs on BMI are less clear. In a retrospective study comparing over 20,000 (twenty thousand) PWLH who initiated an INSTI or a PI-based regimen, those on PIs have significantly lower weight gain than those on INSTIs [98]. On the contrary, in a further retrospective study, the weight increase of previously treatment-naïve PLWH assigned to receive PI- or INSTI-based regimens does not differ between the two groups [99]. Several studies demonstrate that PIs are associated with greater weight increases compared with NNRTIs [60,81]. The effects of the different PIs on metabolism and fat gain seem to vary. Compared to darunavir/ritonavir, the combination of atazanavir/ritonavir leads to significant increases in the total and subcutaneous fat masses [100].

In a study by Munzer et al. comparing weight gain in a cohort of suppressed, treatment experienced patients, switching to DTG-, EVG/cobicistat, RAL, RPV, or boosted DRV containing regimens, it is found that bDRV is the only combination with significantly less weight increase over the whole follow up period compared to DTG [101]. In a sub-analysis of the NEAT022 trial, PLWH on boosted PIs were assigned to either continue with boosted PI regimens or switch to DTG-based combinations. Those switching to DTG regimens witnessed significantly higher weight increase than those on PIs [56].

### 5.4. INSTIs

Among the several antiretroviral drug classes, INSTIs are the most incriminated with increasing weight in PLWH [25,102].

INSTIs have been implicated with significant weight gain in treatment naïve as well as in treatment experienced patients. This effect varies between the different INSTIs with the first-generation regimens, RAL and ELV, leading to less weight gain than the next-generation INSTIs, namely, DTG and BIC [25,94,103,104]. A longitudinal study demonstrates that INSTIs cause weight increase, especially in the first two years of treatment, while no significant weight gain is witnessed after two years of therapy [105]. Likewise, Milic et al. document significant weight gains only in the first two years after switching to INSTI-based treatments [77].

The North American AIDS Cohort Collaboration on Research and Design (NA-ACCORD) study, with over 20,000 participants, evaluated the impact of INSTI on weight gain in treatment naïve patients [81]. PLWH receiving INSTI and PIs as part of HAART gain significantly more weight than participants receiving NNRTIs in a follow-up period of five years. Among the INSTIs, dolutegravir shows the most profound impact on weight increase, followed by RAL and ELV. A meta-analysis with over 3000 patients totally confirms that INSTIs have a greater impact on weight gain than other classes of antiretroviral agents [106]. As for dolutegravir, its impact on weight is confirmed by another study with over 17,000 participants conducted in Kenya [107]. More specifically, treatment naïve patients receiving dolutegravir gain significantly more weight than patients appointed in the NNRTI group. Weight increase is significantly more profound in female patients, in participants being treated for tuberculosis with lower CD4 counts, and in those who are underweight before HAART initiation. The ADVANCE and NAMSAL studies also demonstrate the significant effect of DTG-based therapies on weight compared to EFV-based treatments [82,108], while the AFRICOS study also records significant weight increases with DTG compared to other regimens [109]. Further studies come to the conclusion that among INSTIs, DTG and BIC cause the most significant weight gain in PLWH [103,104]. DTG is followed by BIC, RAL, and ELV in the rank order of probability [103], and the combination of TAF enhances the effects of BIC and DTG [104]. In a population of incarcerated PLWH, DTG is also associated with the greatest weight increase, RAL with the lowest, and BIC and ELV with intermediate weight gains [110]. However, a meta-analysis of 73 studies concludes that DTG-based regimens have similar effect with BIC-, RAL-, and atazanavir-based regimens [94]. INSTIs have similar impact on the weights of children and youth with perinatally acquired HIV in a small longitudinal cohort study by Koay et al. [111].

The impact of INSTIs on treatment-experienced patients issimilar. Lake et al. demonstrate that PLWH switching to INSTI-based therapies experience significant BMI increase [80]. BMI ≥ 30 kg/m^2^ and age ≥ 60 are risk factors for women and age ≥ 60 for men. Similarly, in a retrospective study of patients having virological suppression for at least two years, the switch from EFV/TDF/FTC to an INSTI-based combination is accompanied by significant weight gain [112]. Those changing to DTG witness the greatest weight gain. Weight gain after switching from an EFV to a DTG-containing regimen is also demonstrated in a South African population [113]. This weight increase is accompanied by an increased risk of hypertension as well. Likewise, in the ATHENA cohort, switching to INSTI is associated with a significant weight increase, which is aggravated by simultaneously starting TAF [88]. DTG also leads to significant weight increase in patients who were previously treated with boosted DRV regimens, as a post hoc analysis of the 96-week NEAT-022 trial demonstrates [114]. However, the weight gain is only modest and is not accompanied by a negative metabolic profile.

Interestingly, the preswitch drug combination seems to predict the magnitude of weight gain after switching to INSTIs [115]. In the East African cohort with over 18,000 participants, patients switching from an efavirenz-based therapy to an INSTI therapy experience significant weight increase, whereas those switching from nevirapine do not. Those findings have been confirmed by a meta-analysis, in which baseline HAART is a predictor of post-switch weight gain [116]. Moreover, in further studies evaluating the effects of switching from efavirenz to INSTI-based therapies, weight increase is significantly higher in patients with poor CYP2B6 metabolizer genotypes [117,118]. Persons with CYP2B6 have higher efavirenz concentrations, which explains the smaller weight gain prior to the switch [118]; however, weight increase after the switch to INSTIs seems to be multifactorial [117]. Similarly, in patients switching to BIC or DTG-based combinations, weight gain differs depending on the type of NRTI (TDF or TAF) before switching [119]. Moreover, the effect of INSTIs on weight seems to be limited in patients who switch from non-INSTI treatments to INSTI treatments rather than those who are on long-term stable INSTIs [120].

Not every study confirms the weight-increasing effects of INSTIs and the differential impact of each INSTI on weight. No changes in weight are documented after switching to an INSTI-based therapy, either DTG or RAL, in suppressed patients by Burns et al. [121], and only minor weight increases in women are recorded in patients in Kenya who switch to dolutegravir [122]. Additionally, in a retrospective study, INSTI is not associated with a higher weight increase than other antiretroviral drug classes [123]. In a retrospective observational study of perinatally infected adolescents and young adults, switching to an INSTI-containing combination does not result in significant weight uptake [124]. As the median age of PLWH increases the risk of metabolic syndrome and CVD increases and the influence of INSTIs on older patients receives more attention. In a longitudinal prospective study, the switch of INSTI-naïve patients to DTG-containing regimen does not significantly increase weight in comparison to INSTI-naïve PLWH during a follow-up of over two years [125]. In patients of the Dutch AGEhIV cohort, where white men comprise the vast majority, switching to INSTI-based regimens does not result in significantly greater weight increases than in non-switching patients [126]. In contrary, in exclusively female populations, switching to an INSTI-based treatment leads to significant weight gain compared to non-INSTI treatments [127,128]. This signifies that sex could be a potential risk factor for weight increases in patients receiving INSTIs. In another exclusively female population, Fuller et al. assess the effect of INSTIs on pregnant women with HIV. The researchers conclude that women with a BMI < 25 kg/m^2^ who conceived while on INSTIs have significantly lower rates of excessive weight gain than pregnant women who initiate an INSTI-based treatment during the pregnancy [129].

A plethora of studies have assessed the roles of different antiretroviral regimens on weight gain. The majority of those show that TDF and NNRTIs have weight-losing effects or at least neutral effects on weight, whereas TAF and INSTIs seem to carry the greatest risks of weight increase. Among INSTIs, BIC and DTG have the greatest impact on weight, followed by the rest. As for PIs, the newer regimens seem to have a more neutral effect than INSTIs. The preswitch combination also seems to play a role in the grade of weight gain after switching.

## 6. Contemporary Agents

### 6.1. Bictegravir

The data on the effects of BIC-based regimens as switch therapy on BMI are contradicting. In a multicenter trial by Sax et al., no evidence of increased weight gain when switching to BIC compared to when switching to DTG-based combination has been witnessed [119]. In transgender women, a population at great risk of CVD due to gender-affirming hormonal therapies, the switch to BIC/FTC/TAF does not result in weight gain compared to the baseline weight [130]. However, the majority of patients are already receiving INSTIs, which could have attenuated the effect of BIC. Nevertheless, two studies by Emond et al. demonstrate that initiating or switching to a single-tablet BIC combination (BIC/FTC/TAF) is associated with a greater BMI increase than initiating or switching to a boosted DRV-containing regimen (DRV/c/FTC/TAF) [131,132].

In a real-world cohort study, a BIC-based combination has a similar effect on BMI with the double combination of lamivudine/dolutegravir when used as a switch therapy [133]. Similar findings of the same-sized BIC regimens and LAV/DTG combinations have been witnessed in other real-world studies [134,135].

Interestingly, like other INSTIs, when used as a switch, BIC combinations do not seem to affect weight in older patients [136,137]. PLWHs older than 55 years, recruited from the BICTEL cohort, who switch to a BIC combination do not witness a weight increase [136]. Likewise, in patients over 65 years old, BIC does not affect weight at all [137].

### 6.2. Two-Agent Combinations

In a small retrospective cohort study switching to a two-agent regimen, lamivudine/DTG or lamivudine/RAL has neutral effects on weight and BMI [138]. No differences between the two combinations have been observed. Similarly, the TANGO study detects no significant weight changes between patients who switch to lamivudine/DTG and those who stay on a TAF-based combination, although significant improvements in the metabolic parameters are observed in the dual combination [37,139,140]. In a cohort of virological suppressed patients who switch to RPV/DTG or to a boosted DRV/lamivudine combination, the former has been associated with a significant weight increase, in contrast to the latter [141]. However, both combinations result in similar increases in fat mass.

### 6.3. Doravirine

Doravirine is a novel NNRTI approved for chronic HIV1 infection by the FDA in the form of a triple combination together with TDF and 3TC, and it has been shown to be non-inferior to EFV-based combinations [142]. In a study comparing the effects of DOR/TDF/3TC to a boosted darunavir combination and EFV combination, no significant differences in weight gain have been recorded between the three groups [143]. Further studies confirm the neutral effect of doravirine on body weight [144]. Given the favorable metabolic profile of the DOR combination [145], it could pose an attractive alternative for patients with increased CVD risk; however, more real-life data are needed [146].

### 6.4. Long-Acting Agents

Cabotegravir (CAB)/RPV is a novel, long-acting two-drug regimen that has been approved recently by the FDA for use in PLWH. In Phase 2a HIV Prevention Trials Network Study 077 (HPTN 077), cabotegravir shows no significant weight increase when administered to uninfected persons in the settings of pre-exposure prophylaxis compared to the placebo [147]. However, the evidence concerning weight gain in PLWH due to CAB/RPV is contradictory. Whereas the FLAIR study does not identify any significant difference in weight gain between long-acting CAB/RPV treatment and oral standard treatment with ABC/3TC/DTG in the first 48 weeks [148]., the ATLAS study records a weight increase of 1.8 kg with CAB/RPV compared to 0.3 kg with the oral standard treatment [149]. In a single-center study, switching to the long-acting injectable CAB/RPV resulted in weight loss compared to the patients who remained on oral INSTI-based regimens [150]. More data on the adipogenic effects of cabotegravir combinations are needed to come to a conclusion.

In summary, some of the contemporary treatment options could offer an alternative for patients witnessing weight increase. More specifically, DOR has a neutral effect on weight, while the data on long-acting agents with CAB are rather contradictory. BIC seems to influence weight no more than DTG. Lastly, the two-drug combination seems to have no significant impact on BMI.

## 7. Switch Policy Approach

Based on the cumulative data supporting the HAART-mediated weight gain on PLWHs, a consortium of physicians, public health specialists, and patient activists, among others, have proposed a bundle of recommendations regarding the time to switch antiretroviral regimens on participants in clinical trials depending on changes in weight or BMI [151]. According to these recommendations, in an enrolled patient, if an increase of >10% in baseline weight or an increase in BMI above 30 kg/m^2^ is observed, the current treatment should be re-evaluated and eventually stopped or switched to another antiretroviral combination. However, a PLWH with a baseline BMI of above 30 kg/m^2^ should not be excluded from clinical trials.

Whether weight gain caused by HAART is reversible after the treatment switch and how much time is needed to return to the baseline weight are questions of enormous clinical value. Pohlman et al. report about a patient who witnesses significant weight gain after switching from TDF/FTC/EFV to TAF/FTC/ELVc and their subsequent weight loss after switching back to the initial regimen [152]. A retrospective study reports that switching from TDF to TAF prevents weight gain in the following two years and improves the lipid profile of those patients [153]. In the CHARACTERISE study, which involves patients previously enrolled in the ADVANCE study, changing from TAF/FTC/DTG to TDF/3TC/DTG leads to a significant weight loss in women after one year but not in men [96]. In over 100 PLWHs from the ATHENA Dutch observational cohort who are first switched to TAF and/or INSTI-based HAART and receive treatment for at least two years, TAF and/or INSTI are discontinued to assess the reversibility of weight gain [154]. In the first 12 months post-switch, a weight loss is recorded. However, it does not reach the target of 7% of the weight. A retrospective longitudinal cohort study of over 1000 PLWH does not show any weight loss after INSTI discontinuation [155]. Conversely, another real-world study of PLWH at risk of weight increase, like those of the female sex or of African/Hispanic origin, demonstrates significant weight loss after switching from INSTI to PI-based regimens, thus supporting a switch-off INSTI strategy to attenuate weight increases in those patients [156]. Thus, according to those studies, no rapid and complete reversibility of weight gain, if at all, can be expected, especially after discontinuing INSTI. The SOLAR study assesses the effects of switching from BIC-based regimens to long-acting combinations [157]. Patients receiving TAF/FTC/BIC for a minimum of 6 months are switched to CAB/RPV. However, the weight remains unchanged in both the CAB/RPV and BIC groups.

In conclusion, policy switching seems attractive for PLWH with excessive weight gain. Most studies demonstrate a moderate weight reduction after switching off from INSTIs; however, it is questionable if this change is long-lasting.

## 8. Evaluating and Challenging the Evidence

In 2024, nine experts in the field attempted to review and provide a critical appraisal of the large amount of ambiguous data [158]. Results from major randomized trials of pre-exposure prophylaxis (PrEP), initiation, and treatment switching were included in the review, highlighting the challenges in assessing the role of ART in weight change. The authors underline the fact that body weight in HIV infection is influenced by an interplay of host and environmental factors and question the incrimination of INSTI and TAF for excessive weight gain. The clinical trials included in the review provide strong evidence that agents like DTG, BIC, and TAF have neutral effects on weight gain and that TDF has an attenuating effect, especially when combined with EFV, suggesting an alternative model for the effect of antiretrovirals on weight gain. The changes in weight in PrEP trials of CAB and TAF that are consistent with those in PrEP trial placebo recipients and in comparable general populations argue against the inherent weight-promoting effects of these two agents.

This is underscored by studies like TANGO and SALSA, where removing the TAF agent [38,39,96,159,160,161,162] has merely a minimal impact on weight. BIC not promoting excess weight gain is also based on studies comparing initiation treatments with BIC/FTC/TAF to DOR/ISL and on studies of switching from BIC (SOLAR and MK-8591A-018). Similar changes in weight are detected when continuing BIC/FTC/TAF compared to those switching regimen [163,164]. The neutral impact of DTG on weight gain, when added to a stable regimen, compared to that of a patient who takes a placebo has also been observed [165]. The findings of the ADVANCE trial, where participants (mainly females) were randomized to receive DTG with TAF/FTC gain significant weight, might be based on the fact that participants in the other two arms are assigned to TDF or TDF and EFV. With regards to the weight drop recorded in participants of the ADVANCE study switching from TAF to TDF, clinicians should balance the benefits of TAF compared to TDF in renal and bone integrity and the weight gain before deciding on a regimen change. Future research is mandatory to fully clarify the best choice in the regimen.

Limited data are available supporting antiretroviral switches for weight loss, and treatment guidelines recommend against modifications merely due to increases in weight. In SALSA, TANGO, and SOLAR trials, removing TAF from a regimen while maintaining an INSTI does not change body weight [37,39,162,163,166,167].

The consequences of weight gain and obesity among PLWHs have been assessed [21,168,169] and some studies conclude that there is an association between weight gain when treated with INSTI and/or TAF (even in a PrEP setting) and increases in diabetes mellitus and hypertension incidence [22,170,171,172]. As in the general population, strategies are needed to mitigate these consequences. Adoption of the Mediterranean diet and exercise has been associated with a decline in weight and improvements in lipid, glycemic, and cardiovascular profiles [173,174,175]. A healthier lifestyle should be recommended to all individuals with excessive weight gain. Furthermore, medical or surgical treatment of obesity can also be considered for PLWHs.

Summing up, the latest evidence is reassuring about the impacts of INSTIs and TAF on BMI. The documented weight gains of PLWHs receiving those regimens compared to combinations that include TDF and NNRTIs (EFV) are attributed to the attenuating action of TDF, especially when combined with EFV, rather than to the sheer impacts of TAF or INSTIs. The experts focus on the interplay of host and environmental factors and their impacts on BMI, and they underline the significance of a healthier lifestyle, including a Mediterranean diet and exercise.

The aforementioned studies assessing the impact of HAART on weight are subjected to a series of limitations that should be considered when trying to clarify the HAART-BMI relation. Many of these studies do not take into account other cofounders, modifiable or not, which could influence weight in PLWHs. These include genetic factors which could influence the antiretroviral regimen’s specific effect on metabolism. Some exclusions that are also indicative of the importance of genetic profiling include the study by Erlandson et al. [78], which investigates the role of mitochondrial DNA haplotypes on weight increases, and the studies that assessed the impact of CYP2A6 genotype [117,118]. As for modifiable cofounders, the roles of diet and physical activity as parts of a balanced healthy lifestyle in avoiding and treating excess weight cannot be overstated. Many of the studies mentioned before do not take lifestyle into consideration when assessing HAART’s relation to weight gain. As the studied patient cohorts have significantly diversified cultural and socioeconomic backgrounds, which are reflected especially in diet, the influence on weight could be substantial and variable. Microbiome and enteric dysbiosis play central roles in weight gain in PLWHs, as mentioned before. Nevertheless, this important factor is not routinely investigated when assessing the effects of antiretrovirals on weight. Lastly, preconditions related to weight gain, like depression, menopause, comedication, and lack of physical activity due to medical reasons, are either not or only partially taken into account when assessing the influence of HAART on weight gain.

## 9. Conclusions

In each case of excessive weight increase, clinicians should individualize the data and consider contributing factors, like concomitant drugs (i.e., antidepressants), smoking, endocrine defects, and mood and stress disorders. The time frame of weight gain upon an antiretroviral switch or initiation can also be informative since body weight changes usually occur shortly after starting a new agent. This can be determined by closely observing the rate of weight increase within the first year. In conclusion, a more careful consideration of the available data should be adopted when making HIV treatment decisions. The uptake in weight recorded in individuals treated with newer INTIs and/or TAF in clinical trials and clinical studies or derived as feedback from clinical practice has led to a perception that these agents are causative factors. These observations have led physicians to consider alternative regimens to initiate or switch a patient’s treatment to avoid or reverse significant weight gain. However, a more precise approach to the major clinical trials confirms that TDF-based regimens, especially when combined with EFV, attenuate weight uptake, while BIC, DTG, and TAF should be considered neutral and responsible for excess weight gain. By assessing the efficacy and safety properties of BIC, DTG, and TAF-based ART combinations, one can assume that any association with an uncontrolled or unexpected increase in body weight should be carefully evaluated before being regarded as causal.

## Data Availability

No new data were created or analyzed in this study. Data sharing is not applicable to this article.
